# A Rare Case of Recurrent Ovarian Torsion in a Premenarchal Patient Occurring Twice Within a Two-Month Period

**DOI:** 10.7759/cureus.106405

**Published:** 2026-04-03

**Authors:** Hamsaveni Murday, Varsha Sajeesh, Madeleine Jones, Dinesh Epitawela

**Affiliations:** 1 Department of Obstetrics and Gynecology, Monash Health, Melbourne, AUS

**Keywords:** laparoscopic detorsion, ovarian torsion, pediatric ovarian torsion, recurrent ovarian torsion, simple ovarian cyst

## Abstract

Ovarian torsion is defined as the twisting of the ovary at its pedicle, which can result in significant vascular and lymphatic compromise. This condition is a rare gynecological emergency, warranting prompt recognition and diagnosis. This report describes an 11-year-old girl who presented with a two-day history of left iliac fossa pain and associated vomiting. Ultrasound revealed a 3-cm simple ovarian cyst on the left ovary, with preserved blood flow but evidence of ovarian edema, raising suspicion for ovarian torsion. The patient underwent a diagnostic laparoscopy, which confirmed left ovarian torsion, and a detorsion was performed. Nine weeks later, the patient re-presented with similar symptoms, and intraoperative findings again confirmed left ovarian torsion. At this presentation, left ovarian detorsion and cystectomy were performed. This case underscores the diagnostic challenges posed by the nonspecific presentation of ovarian torsion and highlights the importance of investigating predisposing factors in recurrent cases.

## Introduction

Ovarian torsion is the fifth most common surgical emergency, accounting for approximately 3% of all gynecological emergencies [1]. Ovarian torsion is a high-risk but low-prevalence condition requiring prompt diagnosis and management. It refers to the partial or complete twisting of the ovary at its pedicle, leading to vascular and lymphatic compromise, ischemia, and ultimately necrosis. When twisting phenomena also involve the fallopian tube, they are referred to as adnexal torsion. The majority of ovarian torsion cases are noted in women over the age of 20 years [2]. Here, we present a case of pediatric ovarian torsion that recurred within a short interval and required repeat surgical intervention.

## Case presentation

An 11-year-old prepubertal female patient presented to the emergency department of a tertiary hospital with a two-day history of left iliac fossa (LIF) pain. The pain was acute-onset, twisting in nature, and radiated to the left flank. It was associated with one episode of vomiting and a single loose bowel motion. She had no associated infective symptoms, no significant past medical or surgical history, and had not yet reached menarche.

**Figure 1:**
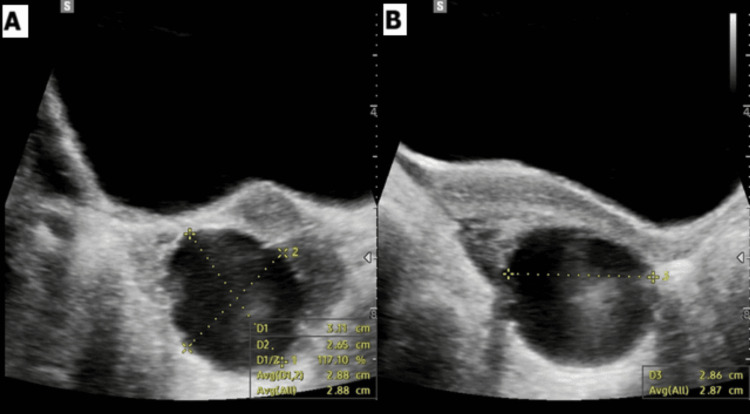
Ultrasound image demonstrating the left ovarian simple cyst in (A) transverse and (B) longitudinal views

On examination, the patient was hemodynamically stable. Abdominal examination revealed a soft, nonperitonitic abdomen with relative nontenderness in the LIF. Pelvic ultrasound demonstrated a 31 × 27 × 29 mm simple unilocular cyst within the left ovary (Figure 1). Doppler interrogation demonstrated preserved ovarian blood flow; however, the left ovary appeared edematous, with findings highly suspicious for ovarian torsion. Laboratory investigations were within normal limits, including normal full blood count, C-reactive protein, and renal function, with a negative pregnancy test supporting the suspected diagnosis of torsion on imaging assessment.

**Figure 2:**
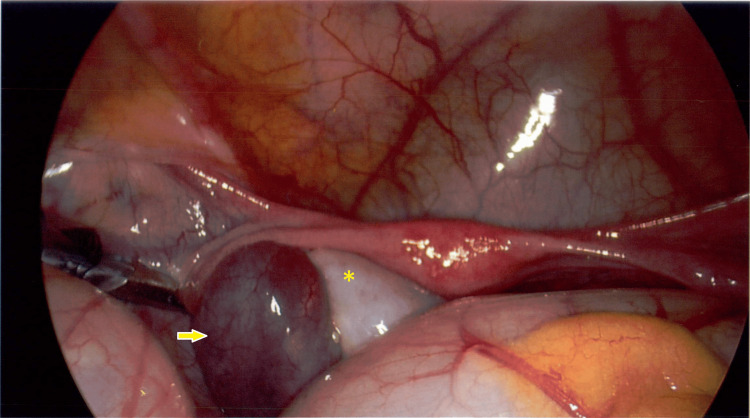
Original intraoperative finding at time of initial laparoscopy of left ovary (*) with simple cyst (arrow)

Given the discrepancy between clinical (nontender abdomen) and ultrasound (edematous ovary findings), discussions ensued between the pediatric surgical and gynecological teams. Given the patient’s age (<14 years; per local protocol), the pediatric surgical team proceeded with diagnostic laparoscopy. Intraoperatively, the left ovary was found to be torted 360° with associated free fluid in the pelvis. The ovary was detorted, and the 3-cm ovarian cyst was left in situ (Figure 2). The patient had an uneventful postoperative recovery and was discharged the following day with a planned clinic review in six weeks, including a repeat ultrasound. A follow-up ultrasound performed four weeks after the initial presentation showed a persistent 3-cm left ovarian cyst but otherwise normal ovaries.

**Figure 3:**
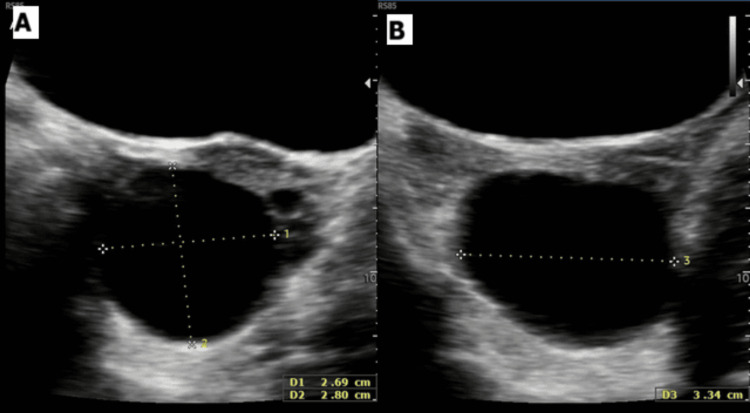
(A) Ultrasound demonstrating stable appearance of left ovarian simple cyst at four weeks post-op. (B) Longitudinal view confirming a thin-walled cyst with anechoic contents with a maximum diameter of ~3.3 cm

The patient re-presented to the emergency department of the hospital nine weeks later with sudden-onset LIF pain, more severe than the initial episode, and an associated episode of vomiting. Pain improved with a small dose of opioids, but was still present, and the patient was able to ambulate post analgesia. A repeat pelvic ultrasound demonstrated a stable 3-cm left ovarian cyst (Figure 3) with no ultrasonographic evidence of torsion and normal vascularity at the time of scanning.

However, due to recurrence of LIF pain and a history of previous ovarian torsion, a decision was made for a repeat diagnostic laparoscopy. Intraoperatively, the left ovary was found to have torted 360° again. The ovary appeared edematous but was not compromised, and the ovarian cyst was removed intact. Additionally, a patent processus vaginalis (PPV) was noted on the left side, and the right side was found to be closed. The patient once again had an uneventful postoperative recovery and was discharged home with follow-up plans in three months. A repeat ultrasound three months later showed normal ovaries, and she was reviewed and discharged from pediatric surgery follow-up.

## Discussion

The incidence of adnexal torsion in female patients younger than 20 years of age is between 4.9 and 30 per 100,000 [2,3]. Notably, 52% of pediatric adnexal torsion cases occur between ages 9 and 14, with a median age of 11 years [2-4]. Our patient presented with recurrent ovarian torsion, an even rarer occurrence in the pediatric population.

Ovarian torsion is seemingly more common in the right adnexa. This could be due to the sigmoid colon on the left, which restricts movement of the left ovary, the hypermobile nature of the cecum on the right, which is more conducive to movement, or because the right utero-ovarian ligament is longer, allowing for more mobility of the right ovary [5-7]. Furthermore, in adults, adnexal torsion is more common in people whose adnexa are distorted by cystic, pregnancy-related, or neoplastic changes. The majority of pediatric cases of torsion (51%-84%) are due to benign cysts (functional, follicular, or hemorrhagic) or ovarian teratomas [2,3]. Interestingly, up to 46% of the pediatric and adolescent cases of adnexal torsion have normal ovaries [2,8]. Predisposing factors include hypermobility of elongated and lax ovarian ligaments, tubal spasms, and hormonal fluctuations in the premenarchal period [2,8].

In this case, a 3-cm left-sided simple cyst, which was not removed during the initial procedure, likely contributed to the recurrence. The cyst may have served as a gravitational pivot, causing an imbalance in ovarian positioning and leading to repeated torsion within a short period of time. Additionally, an incidental finding of a PPV was noted in our patient during the second surgery. In female patients, it is commonly referred to as the Canal of Nuck, with a prevalence of less than 1% in young girls [9]. It refers to the outpouching of the parietal peritoneum through the inguinal ring into the inguinal canal, which can result in cysts and hernias [7]. Canal of Nuck hernias generally present in early childhood (less than five years) but have been reported in ages up to 11 years [7]. Although the presence of a PPV is not a direct predisposing factor for ovarian torsion, in individuals with hypermobile ligaments, the possibility exists that the ovary may herniate into the canal. If the hernia contains the ovary, this increases the risk of ovarian torsion.

The majority of ovarian torsion cases present with sudden-onset, severe unilateral pelvic pain often accompanied by vomiting [5,9,10]. The pain is typically described as constant or colicky and nonmigratory [9]. In cases of intermittent pain, it is imperative to consider torsion and detorsion as a possibility, and it is not uncommon to have a period of intermittent abdominal pain prior to the presentation of ovarian torsion [11]. Fever may also be a presenting finding and is usually associated with the inflammatory reaction accompanying necrosis [11]. A palpable mass may be appreciated, and tenderness may also be present on the ipsilateral side. Given the need for prompt diagnosis and treatment, the diagnosis is challenging, as the presenting symptoms often mimic pathologies in neighboring organs. Acute abdominal pain of this nature gives a broad range of differentials, including but not limited to appendicitis, pelvic inflammatory disease, renal stones, ectopic pregnancy, and diverticulitis [2]. As such, the presentation of a female patient with acute unilateral abdominal pain should always trigger consideration for the possibility of ovarian torsion.

Ultrasound is usually the preferred initial imaging modality. Ultrasonographic findings suggestive of ovarian torsion include a combination of ovarian enlargement, multiple follicles, cyst, whirlpool sign, and decreased or absent color Doppler flow to the affected ovary [7]. Doppler studies in the diagnosis of ovarian torsion are limited by their low sensitivity, with reduced or absent Doppler flow to the affected ovary only being suggestive of ovarian torsion [2]. In cases where a definitive diagnosis cannot be made using ultrasound, computed tomography or magnetic resonance imaging can be used [2,7,11]. None of these imaging modalities can demonstrate torsion-detorsion. Definitive diagnosis is dependent on laparoscopic surgery.

Given the potential for ovarian necrosis and its subsequent impact on fertility, the primary aim of management is ovarian preservation. There is no definite timeframe within which ovarian viability is preserved, with some studies reporting ovarian function preservation up to 72 hours from the onset of symptoms [12].

Laparoscopy, the preferred approach over laparotomy, reduces recovery time and is thought to reduce the risk of adhesions [2]. Traditionally, oophorectomy was performed if the ovary appeared necrotic. However, current evidence favors detorsion over oophorectomy, as it has been shown that detorsion can improve even necrotic-appearing ovaries [2,13,14]. If a simple cyst is present, concomitant cystectomy is preferred as the risk of malignancy in this age group is low [13]. In cases where cystectomy is not done because the risk of damage to the ovary at the time is significant, incision and drainage of the cyst may be considered with serial monitoring via ultrasound. Oophoropexy is a procedure that aims to affix the ovary to limit its mobility. It is not routinely performed after detorsion and is considered only in cases with congenitally long ligaments, recurrent torsion, or a single ovary [15]. There is limited evidence on the long-term risks of oophoropexy, with some studies suggesting possible impact on fertility, atrophic ovaries, and increased risk of menstrual disorders, including dysmenorrhea, dyspareunia, and pelvic pain [16-18]. In the case presented in this paper, oophoropexy was not considered because the recurrence of torsion was thought to be due to the ovarian cyst, which was removed during the second procedure.

The risk of retorsion is increased in those who presented during the initial episode without any adnexal masses [4,19]. A multicenter study reported a recurrence rate of 12% with a risk increase by up to seven times in those who presented initially with idiopathic torsion [4]. Another study found that younger age (<15 years) and smaller cyst diameter (<45 mm) were independent risk factors for recurrent ovarian torsion [20]. It is to be noted that this case report is limited by the single-patient nature and short-term follow-up (three months) of the patient in the postoperative period.

## Conclusions

Ovarian torsion is a rare cause of acute abdominal pain in pediatric and adolescent patients. Its nonspecific presentation, coupled with the potential for significant adverse effects on fertility, underscores the necessity for prompt diagnosis and surgical intervention. Recurrent ovarian torsion is even rarer and warrants investigation for persistent predisposing factors. In this case, the 3-cm ovarian cyst was likely the underlying cause, which was removed during the second surgery, eliminating the need for oophoropexy. This case emphasizes the need for further research into the indications for oophoropexy, both in pediatric and adult patients, to prevent recurrence. Additional studies are required to evaluate the risks and benefits of this procedure and to determine whether its advantages outweigh potential complications.
